# SARS-CoV-2 and Viral Sepsis: Immune Dysfunction and Implications in Kidney Failure

**DOI:** 10.3390/jcm9124057

**Published:** 2020-12-15

**Authors:** Alessandra Stasi, Giuseppe Castellano, Elena Ranieri, Barbara Infante, Giovanni Stallone, Loreto Gesualdo, Giuseppe Stefano Netti

**Affiliations:** 1 Nephrology, Dialysis and Transplantation Unit, Department of Emergency and Organ Transplantation, University of Bari, 70124 Bari, Italy; alessandra.stasi@uniba.it (A.S.); loreto.gesualdo@uniba.it (L.G.); 2 Nephrology, Dialysis and Transplantation Unit, Department of Medical and Surgical Sciences, University of Foggia, Viale Luigi Pinto, 71122 Foggia, Italy; giuseppe.castellano@unifg.it (G.C.); barbarinf@libero.it (B.I.); giovanni.stallone@unifg.it (G.S.); 3 Clinical Pathology, Department of Surgical and Medical Sciences, University of Foggia, Viale Luigi Pinto, 71122 Foggia, Italy; elena.ranieri@unifg.it

**Keywords:** SARS-CoV-2, acute kidney injury, sepsis

## Abstract

Severe acute respiratory syndrome coronavirus 2 (SARS-CoV-2), the causal agent of coronavirus disease 2019 (COVID-19), first emerged in Wuhan, China. The clinical manifestations of patients infected with COVID-19 include fever, cough, and dyspnea, up to acute respiratory distress syndrome (ARDS) and acute cardiac injury. Thus, a lot of severe patients had to be admitted to intensive care units (ICU). The pathogenic mechanisms of SARS-CoV-2 infection are mediated by the binding of SARS-CoV-2 spikes to the human angiotensin-converting enzyme 2 (ACE-2) receptor. The overexpression of human ACE-2 is associated with the disease severity in SARS-CoV-2 infection, demonstrating that viral entry into cells is a pivotal step. Although the lung is the organ that is most commonly affected by SARS-CoV-2 infection, acute kidney injury (AKI), heart dysfunction and abdominal pain are the most commonly reported co-morbidities of COVID-19. The occurrence of AKI in COVID-19 patients might be explained by several mechanisms that include viral cytopathic effects in renal cells and the host hyperinflammatory response. In addition, kidney dysfunction could exacerbate the inflammatory response started in the lungs and might cause further renal impairment and multi-organ failure. Mounting recent evidence supports the involvement of cardiovascular complications and endothelial dysfunction in COVID-19 syndrome, in addition to respiratory disease. To date, there is no vaccine, and no specific antiviral medicine has been shown to be effective in preventing or treating COVID-19. The removal of pro-inflammatory cytokines and the shutdown of the cytokine storm could ameliorate the clinical outcome in severe COVID-19 cases. Therefore, several interventions that inhibit viral replication and the systemic inflammatory response could modulate the severity of the renal dysfunction and increase the probability of a favorable outcome.

## 1. Introduction

The new respiratory infectious disease, coronavirus disease 2019 (COVID-19), first originated in Wuhan, China, and was triggered by a new strain of coronavirus, called severe acute respiratory syndrome coronavirus 2 (SARS-CoV-2). As of November 2020, it has infected 57.531.154 individuals and caused more than 1.370.606 deaths globally [1]. The clinical spectrum of COVID-19 infections ranges from mild symptoms to severe forms. It has been reported that 80% of cases are mild; 10% are severe, developing severe diseases including pneumonia and dyspnea [2]; 2% are critical, and develop respiratory failure, septic shock and multi-organ failure requiring intensive care treatment; and, in about 2% of the overall reported cases, the virus is lethal [2].

The most common clinical manifestations of the infection include fever, cough, myalgia or fatigue, dyspnea, sputum production, and the presence of bilateral infiltrates on chest imaging [2]. In addition, some COVID-19 patients developed acute respiratory distress syndrome (ARDS) [3]. Others have reported acute cardiac injury, acute kidney injury (AKI) [4], and shock, and many of them had to be admitted to the intensive care unit (ICU) [5].

The pathogenic mechanisms of SARS-CoV-2 infection are derived from the comparative genomic analysis of the other two analogous coronaviruses, SARS coronavirus (SARS-CoV) and MERS coronavirus (MERS-CoV) [2]. The genomic analysis revealed that SARS-CoV-2 had a sequence identity of 79% with SARS-CoV and 50% with MERS-CoV, and presented a strong similarity to the pathogenic mechanism of SARS-CoV infection [2,6,7].

Indeed, the new coronavirus, like SARS-CoV, mediated the host cell infection by the binding of SARS-CoV-2 spikes to human angiotensin-converting enzyme 2 (ACE-2) receptors [8].

The ACE-2 is expressed in various human organs due to its pivotal role in a variety of pathological and physiological conditions [9]. Certainly, the expression of ACE-2 on the surface membrane of alveolar cells triggers pulmonary injury, and it is commonly accepted that the lung is the primary target organ of SARS-CoV-2 infection.

The occurrence of AKI has often developed in patients infected by SARS-CoV-2. The pathogenesis of AKI in COVID-19 has a multifactorial etiology [10]. First of all, the expression of ACE-2 has been described in several organs, including the kidney [11]. Several studies reported the expression of ACE-2 in podocytes and proximal straight tubular cells, suggesting that SARS-CoV-2 could directly infect the human kidney and induce cytopathic effects in renal cells, contributing to AKI and the spread of the virus in the body [9,12,13,14].

Another important finding was that COVID-19 patients with more severe clinical manifestations had higher serum concentrations of pro-inflammatory mediators and immune cells dysfunction that enhanced cytokine storm, leading to renal dysfunction, as observed in sepsis disease [15,16,17]. Therefore, AKI may have an inflammatory etiology mediated by an exacerbated immune response.

In addition, kidney dysfunction could hamper the host’s inflammatory response starting at the lung, inducing further renal impairment and the severe damage of the other organs, such as the lungs and heart [18]. In this way, the kidney could lead to a self-amplifying inflammatory response that rapidly increases both the local and systemic damage.

Emerging evidence supports support the detrimental role of endothelial dysfunction and coagulopathy in predisposing to the development of renal dysfunction in COVID-19 syndrome.

Currently, the therapeutic approach is based on supportive care treatment, since vaccines or target therapies are not yet available [2]. Concerning blood purification treatment, it is considered an option for severe cytokine release syndrome (CRS) and to restore immune homeostasis in COVID-19 patients.

Since AKI is a frequent complication in critically ill patients, understanding the principal mechanism of renal impairment could help us to discover new effective therapeutic strategies that are capable of counteracting kidney injury and improving the clinical outcome of COVID-19 patients [2].

## 2. SARS-CoV-2: Mechanism of Cellular Infection

Coronaviruses are single-stranded RNA viruses belonging to the Coronaviridae family. Based on their genomic structure, they are classified into four genera: α, β, γ, and δ. Both α and β coronaviruses tend to infect mammals, and they include SARS-CoV, MERS-CoV, and SARS-CoV-2 [19].

The life cycle of all viruses depend on host cells, and involve five stages—attachment, penetration, biosynthesis, maturation, and release—which are necessary for their replication and metabolic processes. The attachment is the first step in which the virus interacts with specific host receptors and penetrates host cells through endocytosis or membrane fusion. After entering the host cells, the viral RNA is released in the host cytoplasm, and it is used to synthetize viral proteins. The final stage consists of the final maturation and virus release [20].

As is well known, Coronaviruses present four structural proteins: spike (S), membrane (M), envelop (E), and nucleocapsid (N) [21]. Spike protein is a transmembrane glycoprotein, formed by two functional subunits. The S1 subunit is necessary for the binding of the host cell receptor, and S2 is important for the penetration stage. As for SARS-CoV infection, human ACE-2 (hACE-2) was identified as the functional receptor that is responsible for the binding of SARS-CoV-2 to host cells [22]. After the host’s exposure to SARS-CoV-2, the viral entry is dependent on the interaction between the NH-terminal peptidase domain of ACE-2 at the surface of the host cells and the viral S1 domain [23]. Then, the S1 domain is cleaved by host furin-like protease, inducing conformational changes of the S2 site that facilitate the fusion of the viral and host cellular membrane. Moreover, the low pH and pH-dependent endosomal cysteine protease are also required to mediate the virus penetration in the host cells [24]. After entering into the cytoplasm, the viral RNA is translated in its replicase and structural proteins through the host transcriptional machinery, generating new virions and extending the site of infection [24].

The ACE-2 is an important regulator of the renin–angiotensin system (RAS) that has a crucial role in the balance between fluid and salts, and in the homeostasis of blood pressure. In addition, RAS controls local cellular responses, physiological developmental processes and organ function [25]. In RAS, ACE-2 cleaves Angiotensin (Ang) II, which mediates vasoconstriction pro-inflammatory and pro-fibrotic process, and converts it into Ang (1–7), which has opposite effects, promoting vasodilation and anti-proliferative pathways [25]. Then, ACE-2 regulates the physiological and pathological mechanisms of different organs [25].

Several studies have underlined the involvement of ACE-2 in the pathological progression of several diseases, and it may also be implicated in the susceptibility to SARS-CoV-2 infection and the clinical outcome of COVID-19 patients [26]. Indeed, the ACE-2 has been specifically reported in type II pneumocytes, and functions as a receptor for SARS-CoV-2, suggesting that the lungs are the primary target for the virus attack [22]. Moreover, it has been found in various organs, including the kidneys, demonstrating that SARS-CoV-2 could directly infect other organs in addition to the lungs [27].

Recently, Shang et al. deeply described the cell entry mechanism of SARS-CoV-2, which explained its potency and evasiveness, and the differences between SARS-CoV-2 and SARS-CoV infection [22]. First of all, the SARS-CoV-2 receptor binding domain (RBD) showed a significantly higher hACE-2 binding affinity than the SARS-CoV RBD [22]. In addition, its RBD was mostly in the lying-down state [28,29]; this ineffective state in hACE2 binding was a strategy to maintain RBD that is less accessible, in order to evade immune surveillance. Interestingly, SARS-CoV-2 applied a second strategy to assure its high infectivity through host protease activation [22]. Given that there is an extra genetic region unique to SAR-CoV-2 that translates to a furin-like cleavage site in the S protein [30], the authors showed that furin pre-activation was necessary for the virus entry into several hACE2-expressing cell lines [22]. Moreover, they observed that transmembrane serine protease 2 (TMPRSS2) and lysosomal cathepsins were also required to process the SARS-CoV-2 S protein, and facilitated host cell entry [22].

Since the RBD of SARS-CoV-2 is less accessible in the lying-down state, the efficacy of vaccines based on neutralizing antibodies for RBD may be limited; this approach may be hardly successful. Considering that SARS-CoV-2 uses several proteases for cell entry, the use of inhibitor mixtures against multiple protease activators and siRNA approaches could counteract the infectivity of the virus and achieve a satisfactory outcome.

## 3. Kidney Dysfunction in SARS-CoV-2 Infection and the Risk for Progression to Chronic Kidney Disease

The lungs are the primary organs affected by SARS-CoV-2 infection; however, recent evidence has demonstrated that AKI, cardiac damage and abdominal pain are the most common co-morbidities observed in people severely affected by the COVID-19 disease [31].

The exact incidence of AKI in COVID-19 disease is still not fully clear [32]. Huang et al. reported that 7% of SARS-CoV-2 infected patients developed AKI, and 23% of critically ill patients experienced AKI [33]. In another study conducted on 99 patients affected by COVID-19 disease, seven cases developed various levels of renal damage, and three of them were diagnosed with AKI [34]. In addition, in a multicenter study, Guan et al. reported that the AKI incidence rate was only 0.5% [35].

In a recent study conducted on 59 SARS-CoV-2 patients, Li et al. demonstrated that a massive albuminuria occurred in 34% of subjects on the first days of the admission, and 63% of them developed proteinuria during their hospitalization [36]. In addition, blood urea nitrogen (BUN) was increased in 27% overall, and—more interestingly—two thirds of the deaths were correlated with both elevated BUN and serum creatinine (SCr) over 200 µmol/L [36]. Recently, Cheng et al. showed that 44% of hospitalized patients with COVID-19 presented proteinuria and hematuria, and 26.7% overall had hematuria on admission. Moreover, the increasing rates of BUN and SCr were 14.1% and 15.5%, respectively, and the incidence of AKI was 3.2% [37]. Finally, this study reported that 50% of the patients who developed AKI, died [37]. Therefore, it appears that the incidence rate of AKI in SARS-CoV-2 infection is lower than in SARS and MERS disease.

However, Yang et al. recently reported that, amongst critically ill hospitalized patients with COVID-19 in Wuhan, China, 29% of them were affected by AKI [38]. Accordingly, Diao et al. [13] demonstrated in a retrospective study that 27.06% of patients had AKI, and that older patients had a higher risk to develop AKI [13]. Therefore, there is a high incidence of AKI in patients with severe and critical conditions.

Moreover, in a retrospective study conducted on 163 critically ill patients who recovered from SARS-CoV-2 infection, only one subject presented AKI; instead, 28 patients out of 113 critically ill patients who did not survive developed AKI during the course of their hospitalization [39]. Clearly, AKI plays an important role in the prognosis of critically ill SARS-CoV-2 patients, and it is associated to a high incidence of death [31].

Clearly, in order to understand the incidence of AKI in COVID-19 disease, it is is important to consider the demographic risk factors and clinical conditions of patients at admission and during hospitalization [10]. Recent data proposed several demographic parameters—such as male sex, older age, diabetes mellitus, hypertension, cardiovascular disease, congestive heart failure, high body mass index, chronic kidney disease (CKD), genetic risk factors, immunosuppression, and smoking history—that could induce or increase the incidence and progression of AKI [10,40,41,42]. Lin et al. performed the first systematic analysis of the COVID-19 risk factors on 79 research articles, and showed that patients with advanced age were more vulnerable to the development of AKI, probably due to weakened immune system function and the physiological aging of organs [43]. Moreover, Shastri et al. observed that male patients had a lower capacity in SARS-CoV-2 clearance after infection compared to female patients, underlying the gender disparity in the disease’s severity [44]. Moreover, the impact of high-risk behaviors like smoking and alcohol consumption could make male patients more susceptible to AKI in COVID-19 disease [43]. Moreover, certain genetic traits might increase susceptibility to the development of AKI in the course of SARS-CoV-2 infection [10]. Several reports have revealed histological signs of collapsing glomerulopathy in African American patients with COVID-19 infection [45,46,47]. As is well known, the Apolipoprotein L1 (APOL1) risk allele is more common in African people, and confers a high risk of renal injury to this ethnic group [48]. Therefore, it is plausible that collapsing glomerulopathy following SARS-CoV-2 infection may be associated with APOL1 kidney risk alleles [10]. Otherwise limited data suggest that polymorphisms in the ACE-2 gene might influence the ability of the virus to infect cells [10]. The mortality rate and incidence of AKI vary considerably between countries and different health care systems [10]. Data from several studies in China showed that AKI is less common among COVID-19 patients compared to patients in the USA and Europe [33,35,49,50]. Indeed, Chinese patients had fewer comorbidities, and were admitted to hospital with less severe respiratory disease. To our knowledge, there are no relevant results that correlated the different hospital settings and the risk of AKI [33,35,49,50]. Furthermore, the clinical conditions of patients at admission—such as the severity of COVID-19, dehydration, hypoxemia, lymphopenia, leukocytosis, increased markers of inflammation, rhabdomyolysis and medication exposure with ACE inhibitors and/or angiotensin-receptor blockers (ARBs), statins, nonsteroidal anti-inflammatory drugs and other potentially nephrotoxic drugs—may induce renal damage [10]. Among hospitalized COVID-19 patients, those exposed to mechanical ventilation, nephrotoxins like antibiotics or contrast media, and vasopressors as part of their clinical care, had a high incidence of AKI and experienced worse outcomes [10]. Therefore, as reported in the consensus report of the 25th Acute Disease Quality Initiative (ADQI) Workgroup [10], clinical risk stratification is necessary in order to prevent the occurrence of AKI in COVID-19 disease, and to improve the treatment strategies in order to assure the better prognosis of critically ill patients.

Since several clinical and experimental studies have shown that the severity of AKI is strongly associated with the development of fibrosis and the progression to chronic kidney disease (CKD) [51,52], it is reasonable to expect the same scenario in an increased load of patients with COVID-19–associated AKI. Recently, Chan et al. published a clinical study report about the persistence of kidney dysfunction in survivors of COVID-19–associated AKI [53]. This study analyzed 3993 hospitalized patients with COVID-19 in New York City, and showed that a third of the patients with AKI had not recovered to baseline kidney function at the time of discharge [53]. The low recovery rate is expected to be due to the severity of the AKI in these patients. Indeed, several studies in many patients with COVID-19 have found remarkable signs of damage in tubular cells and podocytes associated with proteinuria, hematuria or leukocyturia, and microthrombi [14]. Similar results were also found in a Chinese cohort study, with less than half of the patients recovering their kidney function [41]. The delayed consequences associated to AKI and the risk of CKD progression remain to be elucidated. From a pathophysiological point of view, the detrimental events that characterize COVID-19–associated AKI—such as endothelial dysfunction, hypoxic insult, immunological dysregulation, inflammatory processes, tubular damage and the impairment of parenchymal repair—could trigger the transition process to CKD. Therefore, further investigations are required on the clinical follow-up of post-AKI patients in a COVID-19 setting.

## 4. Pathogenesis of AKI in COVID-19 Disease: Possible Role of ACE-2 and Direct Viral Infection

**Figure 1:**
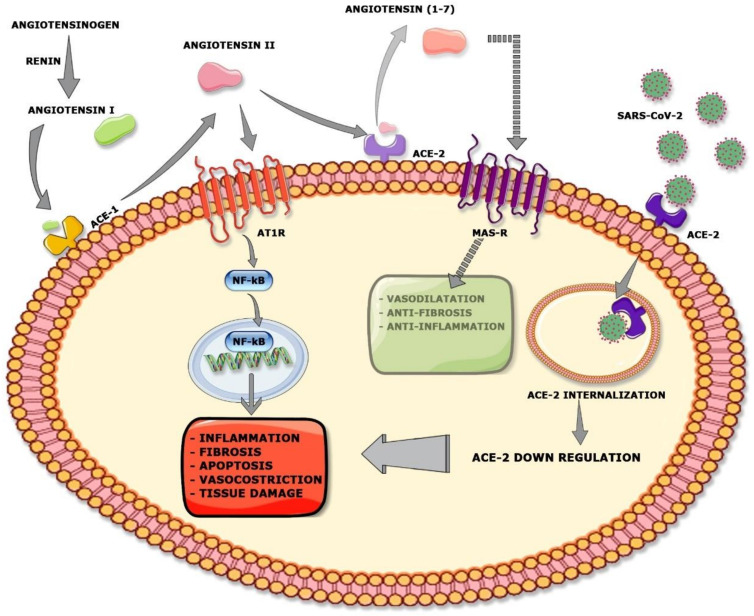
Viral entry mechanism of SARS-CoV-2 and RAS impairment. Angiotensinogen is cleaved into angiotensin I by renin, and then into angiotensin II by ACE-1. Then, Angiotensin II binds angiotensin receptor type I (AT1R) and modulates the gene expression of several inflammatory cytokines via NF-κB signaling, inducing deleterious effects (i.e., inflammation, fibrosis, apoptosis, vasoconstriction, and tissue damage). In normal conditions with a balanced RAS, both ACE-1 and ACE-2 are activated. Therefore, ACE-2 counteracts AT1R activation, inducing the cleavage of angiotensin II into angiotensin 1–7. Angiotensin 1–7 interact with the MAS receptor (MAS-R), promoting vasodilatation, and anti-fibrotic and anti-inflammatory processes. SARS-CoV-2 enters the host cell by binding with the ACE-2 receptor. The virus endocytosis causes a decrease of ACE-2 expression on the surface membrane that results in a down-regulation of the MAS-R pathway (faded image). Moreover, the accumulation of Angiotensin II induces an overactivation of AT1R signaling, increasing the cell damage.

Our current knowledge on the pathogenesis of SARS-CoV-2–induced AKI is largely presumptive, and comes from previous studies in MERS and SARS-CoV diseases [32]. Renal dysfunction could be the result of both direct coronavirus infection and the cytokine storm due to abnormal host immune response [32] (Figure 1).

As has previously been underlined, ACE-2 is the main binding site for SARS-CoV-2, and it is not only expressed in lung tissue, but rather has been reported in other organs, including the kidney [54]. In particular, ACE-2 is mainly detected in proximal tubules, on the brush border apical membrane, where it colocalizes with ACE and maintains the homeostasis of the RAS as a negative regulator [26]. It is also present in podocytes, mesangial cells, the parietal epithelium of Bowman’s capsule, and the collecting ducts [11,55,56,57].

The expression level of ACE-2 in the kidneys is crucial in the pathogenesis and progression of renal dysfunction in several diseases [58]. Accumulating evidence has shown that the decrease of ACE-2 expression and activity was implicated in different models of both acute and chronic kidney diseases, and it was associated with the loss of RAS homeostasis and worsened pathological changes in renal parenchyma [58,59].

SARS-CoV-2 may induce renal impairment by different mechanisms, including direct viral infection by ACE-2 binding. It is plausible that the higher renal tropism of SARS-CoV-2, due to the increase affinity of the S1 domain for ACE-2, could allow it to direct the cytopathic effects of the virus and the local disruption of RAS homeostasis [4] (Figure 1).

Indeed, the presence of SARS-CoV-2 RNA and the shedding of viable SARS-CoV-2 in the urine of COVID-19 patients indicates that the virus has a direct interaction with the renal tubules [60]. Moreover, SARS-CoV-2 viral particles have been found in kidney samples from autopsied patients [14,61].

Recently, Diao et al. demonstrated that SARS-CoV-2 induced acute tubular necrosis by direct cytopathic effects [13]. The analysis of renal biopsies revealed the presence of viral nucleoprotein (NP) antigens in in the cytoplasm of tubular cells [13]. Su et al. showed the presence of viral particles in tubular cells and podocytes [14]. It is reasonable that the virus could infect renal parenchyma by first invading podocytes and then binding ACE-2 in the proximal tubule [14].

The principal hallmark of renal dysfunction in patients with COVID-19 is mild or moderate proteinuria [62]. As is well known, a little fraction of plasma protein is filtered in the renal glomeruli, and most of them are reabsorbed by proximal tubular cells. The integrity and functionality of the glomerular filtration barrier depends on its three components: endothelial cells, the glomerular basement membrane, and podocytes [63,64,65]. The involvement of podocytes in COVID-19 disease has been recently determined, and it is plausible that the increased proteinuria is the result of direct cell infection and changes in RAS homeostasis [66]. Indeed, if a pathological process induces an alteration of RAS homeostasis and an increase of Ang II levels, podocytes acquire a dysfunctional phenotype, resulting in single nephron hyperfiltration and increased proteinuria [67].

Certainly, tubular damage can also augment proteinuria, usually with a mild intensity [62]. However, there is no evidence that supports that the alterations of the RAS system could influence tubular behavior, determining the decrease of the protein adsorption.

In lung cells, the viral entry is mediated by ACE-2 and the viral S protein priming serine protease TMPRSS2 [24], whereas renal proximal tubular cells express low levels of TMPRSS2 [68]. Interestingly, several studies have shown that renal cells present elevated levels of other proteases, including cysteine protease cathepsin B/L, glutamyl aminopeptidase, and serine protease dipeptidyl peptidase 4 (DPP4), which might facilitate the binding of SARS-CoV-2 to ACE-2 and viral entry [31]. These potential cofactors play a critical role in SARS-CoV and MERS-CoV infection, and may play an important role in the internalization of SARS-CoV-2 [31].

However, neither of the recent studies have unequivocally demonstrated the direct viral entry in renal cells and consequent viral replication and cytopathy in COVID-19 patients [69]. Therefore, cohort studies on renal biopsies that include a large number of patients with COVID-19 are needed in order to definitively assess the scientific evidence of direct viral tropism.

## 5. Role of Immune Response in SARS-CoV-2–Associated AKI

**Figure 2:**
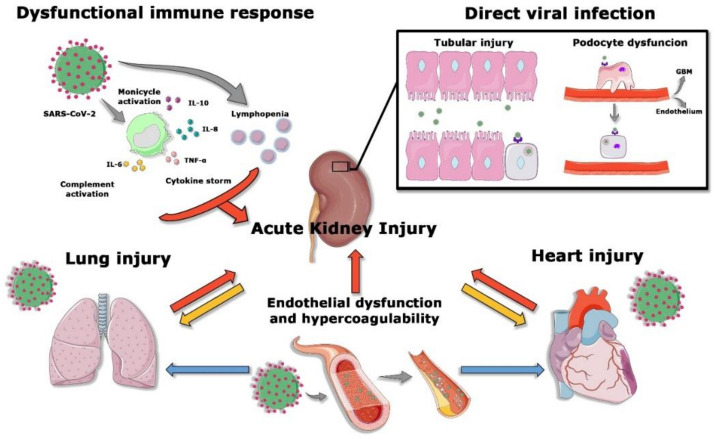
Etiology of AKI in SARS-CoV-2 infection. Several mechanisms contribute to AKI development in COVID-19 patients. The dysfunctional immune response characterized by the production of various proinflammatory cytokines, the complement system activation, and the T cell impairment lead to renal injury. On the other hand, SARS-CoV-2 penetrates tubular cells and podocytes, leading to acute tubular necrosis and podocyte dysfunction. The endothelial damage and the activation of the coagulation system participate in the development of AKI. Moreover, both lung and heart injury affect renal function. In the same way, the kidney hampers lung and heart dysfunction.

The pathogenesis of SARS-CoV-2-induced AKI is multi-factorial (Figure 2). Both direct viral damage and the host immune response are likely to contribute to kidney injury in patients with SARS-CoV-2 infection.

Certainly, SARS-CoV-2 infection is the principal trigger for COVID-19 disease, but the dysfunctional host immune response is considered to be the major player in the progression of the severe disease [70].

Clinical evidence suggests that this response is divided into two phases [71]. During the first phase, SARS-CoV-2 infects the lung epithelial cells of the distal airways, which highly express ACE-2 on the apical surface membrane [27,70,72]. Thus, the infection and the subsequent replication of the virus induces the apoptosis/pyroptosis of the host cells with extensive tissue damage and vascular leakage [70]. Pyroptosis is a highly inflammatory form of programmed cell death triggered by cytopathic viruses, such as SARS-CoV-2 [70]. In most cases, this leads to a local inflammatory response through the recruitment of immune cells from the blood circulation into the infected site, which eradicates the pathogen. Then, as the immune response decreases, the patients recover [70].

However, in some cases, the general state of health, the genetic background, the prolonged presence of viral antigens, and the continued release of pro-inflammatory factors and damage-associated molecular patterns (DAMPS) from damaged or dying cells cause an exacerbated immune response that induces the progression of the disease [70,71]. Therefore, the local immune response turns into a systemic inflammatory response, also termed cytokine release syndrome (CRS), that leads to acute lung injury and ARDS, with detrimental effects across all organs and subsequent multi-organ dysfunction [70].

It remains unclear whether the disease’s progression is related to ongoing virus replication. Several studies have shown that the viral replication and titres in respiratory tract samples are on the decline even before the beginning of critical illness signs of pneumonia both in SARS-CoV and SARS-CoV-2 infection [73,74,75]. However, Zhou et al. showed that viral RNA was still detectable in non-survivors up until the point of death, demonstrating that the failure of the immune response in eradicating the viral infection is another hallmark of poor outcomes [76].

## 6. Viral Sepsis: Cytokine Storm and Multiorgan Dysfunction

The host response to viral infection, including the production of various proinflammatory cytokines and the activation of T cells, CD4^+^ and CD8^+^ T cells, is necessary to control the viral replication and diffusion [77]. In addition, the tissue damage caused by the cytopathic virus amplifies the inflammatory response through the local recruitment of immune cells that release high levels of pro-inflammatory cytokines. The secretion of multiple cytokines, also termed CRS, leads to further tissue injury [78]. As in severe sepsis, the cytokine storm plays an important role in the immunopathology of COVID-19 [79,80].

Different levels of pro-inflammatory cytokines and chemokines were observed in COVID-19 disease from the mild to the severe stage [78]. A retrospective analysis, performed in SARS-CoV-2 infected patients, showed higher levels of the expression of IL-1β, IL-1RA, IL-7, IL-8, IL-10, IFN-ɣ, monocyte chemoattractant peptide (MCP)-1, granulocyte-colony stimulating factor (G-CSF), macrophage inflammatory protein (MIP)-1A, MIP-1B, and tumor necrosis factor-alpha (TNF-α). In addition, higher levels of IL-2, IL-7, IL-17, IL-10, MCP-1, MIP-1A, and TNF-α were found in ICU patients [81].

Yang et al. showed that the severity of lung injury was strongly associated with increased plasma levels of IL-1α, IL-1ra, IL-2, IL-7, IL- 10, IL-17, IFN-ɣ, inducible interferon protein (IP)-10, and G-CSF, which are positively related to SARS-CoV-2 viral titres [82].

Among all of the cytokines, IL-6 plays an important role in CRS. The serum levels of IL-2 Receptor (IL-2R) and IL-6 are positively correlated with the severity of COVID-19 disease [83]. In other studies, the increase of IL-6 was found in mildly- and severely-ill patients. However, Wang et al. found that the pulmonary infiltration area, the damaged lung area, is closely associated with increased levels of IL-6 [84].

Interestingly, increased serum levels of IL-6 are also associated with worse outcomes in AKI, and may be considered to be a good biomarker for the early diagnosis and prediction of clinical outcomes such as the need for dialysis, and mortality [85].

Several clinical and experimental studies have demonstrated that the local activation of IL-6 is implicated in renal autoimmune and inflammatory diseases [85]. Moreover, renal resident cells, including podocytes, endothelial cells, mesangial cells, and tubular epithelial cells can secrete IL-6 under certain conditions [85]. In the meantime, all of these cells, as well as immune and inflammatory cells actively respond to IL-6, and contribute to the exacerbation of renal damage in acute and chronic renal injury [85]. More importantly, the serum increase of IL-6 in AKI has been proven to have a strong correlation with higher alveolar–capillary permeability and pulmonary hemorrhage [86]. In addition, lung damage may also cause renal medullary hypoxia that subsequently contributes to acute tubular necrosis [87]. Therefore, lung–kidney crosstalk is characterized by bidirectional damage [87].

Recently, it was found that IL-6R–neutralizing mAb tocilizumab ameliorates the clinical response in kidney transplant recipients with severe SARS-CoV-2 infection [88].

The importance of IFN-γ has been recently explored in COVID-19 disease [89,90]. Hu et al. demonstrated that the progression of lung injury and fibrosis was inversely related to IFN-γ serum levels in patients with severe SARS-CoV-2 infection [89]. As is well known, IFN-γ is a pleiotropic cytokine with anti-fibrotic activities, and several studies have reported its potential effectiveness in the prevention of fibrosis in renal parenchyma [89,91,92]. Moreover, lower levels of IFN-γ are associated with dysfunctional immune response [93,94,95] and renal tubulointerstitial damage [96] in chronic kidney disease progression [97]. Therefore, IFN-γ could be considered a good biomarker for the prognosis of severe forms of COVID-19 disease, and its potential therapeutic value needs future investigations.

The heart–kidney axis could also contribute to AKI in patients with COVID-19 [98]. It was found that the combination of both decreased cardiac output and acute viral myocarditis induces renal vein congestion, hypotension and subsequent hypoperfusion, lowering the glomerular filtration rate [98]. In addition, AKI developed, on average, nine days after admission, along with secondary infections and acute cardiac damage [99]. On the other hand, the kidney is an early responder to the myocardial dysfunction, and it could lead to hypovolemic shock, with detrimental effects on heart and lung function [87,100].

Taken together, these results indicate that the pathogenetic mechanisms of COVID-19 disease reflect the maladaptive host immune response observed in sepsis, and the advances in the septic field could have a great impact in therapeutic options [78].

## 7. Immune Cell Response Against SARS-CoV-2 Infection

During viral infection, there is a significant increment of neutrophils, leukocytes, and the neutrophil–lymphocyte-ratio (NLR) in severe forms of COVID-19 compared to mild cases. Several studies have reported low levels of T cells in the peripheral blood of patients with the severe disease [81,101]. This condition of lymphopenia suggested that the T cells moved to local infected site to resolve the infection. Moreover, patients with the severe disease presented increased plasma levels of proinflammatory cytokines, including interleukin IL-6, IL-10, G-CSF, MCP1, MIP1α, and TNF-α [81,101]. Interestingly, both the CD4^+^ and CD8^+^ T cells were hyperactive in these patients, as shown by the increased expression of CD69, CD38 and CD44 on their surface membrane. However, Zheng et al. demonstrated that T cells expressed a higher percentage of checkpoint receptor Tm3^+^PD-1^+^, suggesting that the T cells were also exhausted [102]. The exhaustion of antiviral CD8^+^T cells in COVID-19 syndrome was also demonstrated by the expression of NK group 2 member A (NKG2A), another marker for lymphocyte breakdown [102]. This condition was associated with a worse outcome in COVID-19 patients [102].

Another relevant finding was the increased co-expression of IFN-γ and granulocyte-macrophage colony-stimulating factor (GM-CSF) in the CD4^+^ T cells of COVID-19 patients with severe disease progression [101]. GM-CSF is released upon the virus infection, and it plays a critical role in innate immune cell differentiation and T cell function, and can also augment local damage [103].

So far, it remains unclear whether the T cell impairment in COVID-19 disease is due to a direct T cell infection or an indirect mechanism mediated by SARS-CoV-2, such as altered antigen-presenting cell (APC) function, since APCs are important for T cell activation. In addition, T cells are important for the control and counteraction of the overactivation of the immune response during viral infection [104,105]. Therefore, during SARS-CoV-2 infection, the T lymphopenia could aggravate local and systemic inflammation, and multi-organ damage. Accordingly, Liu et al. observed that the T cell count’s reduction was reversely correlated with the kinetic changes of most of the examined cytokine levels in severe COVID-19 patients [106]. When the T cell count dropped down after 4–6 days from the beginning of the disease, the serum levels of IL-10, IL-2, IL-4, TNF-α and IFN-γ significantly increased, while the recovering of the T cells number was associated with the decrease of IL-6, IL-10, IL-2, IL-4, TNF-α, and IFN-γ levels. Therefore, the lymphocyte count may bear a prognostic impact for COVID-19 patients [106].

**Figure 3:**
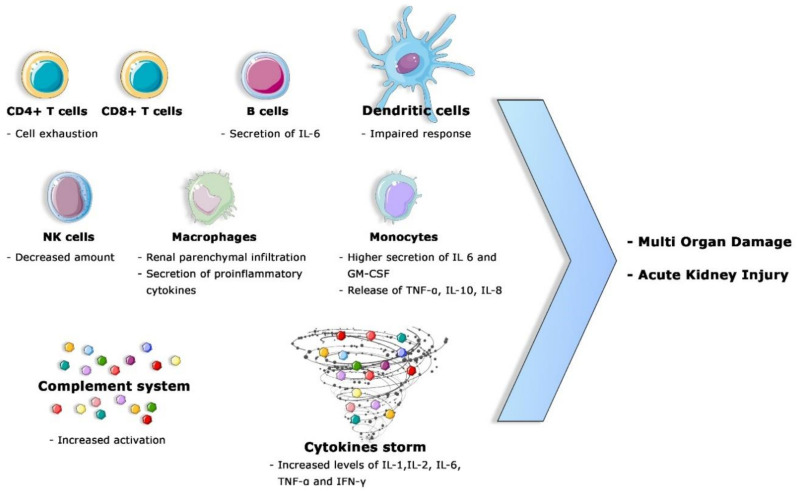
Principal mechanisms of dysfunctional immune response associated with multi-organ damage and AKI. The most important are T cell exhaustion, a decreased amount of NK, dysfunctional B cell activation, impaired DC response, the higher activation of circulating monocytes, the infiltration of macrophages into the renal parenchyma, the higher secretion of pro-inflammatory cytokines (cytokine storm) and systemic complement activation.

In particular, the patients with a severe progression of COVID-19 disease presented remarkably lower levels of helper T cells and regulatory T cells. As is well known, the regulatory T cells are involved in the maintenance of the immune homeostasis through the suppression of the activation, proliferation, and proinflammatory function of most lymphocytes, including CD4^+^T cells, CD8^+^T cells, NK cells, and B cells [107,108]. In addition, the percentage of naïve helper T cells is augmented while the number of memory helper T cells and CD28^+^ cytotoxic suppressor T cells declines in severe COVID-19 disease [81,109]. Therefore, the loss of the equilibrium between naïve T cells and memory T cells impacts the efficacy of the host immune response [110]. In addition, Wang et al. highlighted a strong relationship between inflammatory markers—including ESR, CRP and IL-6—and the lymphopenia [111]. (Figure 3).

Another important finding was that, during SARS-CoV infection, lung epithelial cells produced IL-6 and IL-8 [112]. As is well known, IL-8 is a chemoattractant factor for neutrophils and T cells. Since a large number of immune cells were found in the lung tissue of patients with COVID-19 disease, lung epithelial cells may actively contribute to immune cell recruitment by the synthesis and the release of several chemoattractant factors [113,114].

Several pieces of evidence suggest that IL-8 has a central role in renal interstitial inflammation. Upon proinflammatory stimulation, renal cells are able to synthetize IL-8, and to increase the recruitment of neutrophils and lymphocytes at the tubule-interstitial level [115,116]. In addition, Tang et al. showed that increased levels of albumin and proteinuria could also stimulate the tubular production of IL-8, and it is plausible to hypothesize that similar mechanisms could be observed in SARS-CoV-2- induced AKI [117].

Recently, Zhou et al. showed that the bronchoalveolar fluid (BALF) from patients with severe COVID-19 presented elevated levels of CCL2 and CCL7, two chemokines that are involved in the recruitment of CC-chemokine receptor 2-positive monocytes [118]. Moreover, Liao et al. demonstrated that the mononuclear phagocyte (MNP) compartment was strongly increased in patients with severe COVID-19 compared to patients with the mild disease or healthy controls [119]. In addition, the MNP compartment was principally characterized by inflammatory monocyte-derived macrophages [119]. The inflammatory behavior of these macrophages is in line with the results obtained by RNA-seq in the study of Zhou et al. [118,119]. Moreover, a subset of these macrophages expressed genes involved both in inflammatory and fibrotic processes [118], such as those observed in liver cirrhosis [120]. Therefore, the invasiveness and the pathogenicity of these infiltrating macrophages could be correlated not only to acute inflammation but also to the lung fibrotic damage that was observed in patients under mechanical ventilation [121].

Although the host immune cells infiltrate the infected tissue in order to counteract viral replication, a dysfunctional immune response could induce increased renal damage and promote fibrosis, cell apoptosis, and endothelial dysfunction. Indeed, renal biopsies of SARS-CoV-2 patients presented the infiltration of high levels of CD68^+^macrophages into the tubulo-interstitium, indicating that the proinflammatory cytokines derived from macrophages could increase renal damage [13]. In addition, CD8^+^ T cells were found only in restrained numbers, while CD4^+^ T cells and CD56^+^ NK cells were observed in great amounts [13].

Moreover, a significant expansion of CD14^+^ CD16^+^ inflammatory monocytes was observed in the peripheral blood of COVID-19 patients with severe pulmonary syndrome from ICU compared with those patients who did not require ICU hospitalization [101,122]. These circulating monocytes responded to granulocyte macrophage-colony stimulating factor (GM-CSF) released by pathological T cells, and initiated tissue damage. Interestingly, they were also able to synthetize and secrete GM-CSF and IL-6 [101,122]. Therefore, the significantly higher secretion of IL 6 and GM-CSF in ICU patients induced an inflammatory storm with deleterious lung damage and an increased mortality rate [101].

The role of B cells in SARS-CoV-2 immune response is more controversial [123]. Given that only patients with agammaglobulinemia had a mild course of COVID-19, B lymphocytes could accelerate the inflammatory process [123]. The role of inflammation in aggravating the clinical conditions of COVID-19 patients is well known and has been confirmed in several studies. B cells are able to produce IL-6, which induces germinal center formation and increases the level of inflammation. In an experimental animal model, the decrease of B-cell–derived IL-6 counteracts the spontaneous autoimmune germinal center formation, preventing systemic autoimmunity [124]. In addition, the role of B cells in inducing lung inflammatory damage is also underlined by the presence of granulomatous lymphocytic interstitial lung disease in patients with common variable immunodeficiency that could be treated by B-cell–depleting drugs [125]. Therefore, the dysfunctional immune response to SARS-CoV-2 infection could be counteracted by modulating the B cell response and blocking the release of inflammatory cytokines by monocytes and dendritic cells [123].

## 8. Complement System Activation and Renal Implications

Emerging evidence suggests that the complement system plays a key role in the pathogenic mechanisms of SARS-CoV-2 infection [126]. As is well known, the complement system is the first host immune response that protects against infectious agents such as viruses [127]. However, the exacerbated activation of the complement system can increase the inflammatory response, coagulation process, and cell lysis, leading to local severe tissue damage and systemic inflammation associated with multiple organ failure [51,128,129].

Recently, Diao et al. analyzed renal biopsies from six patients with severe COVID-19, and observed abundant complement deposition on renal tubular cells associated with tubular necrosis and acute renal failure, demonstrating that SARS-CoV-2 infection contributes to complement activation and renal damage [126]. In addition, Gao et al. demonstrated the deposition of several complement factors—such as mannose-binding lectin (MBL), C4, C3 and C5b-9—on the surface membrane of different cells of the lung parenchyma [126,130]. Interestingly, they observed that SARS-CoV-2 N protein enhances mannan-binding lectin serine protease 2 (MASP-2) activity and increases C4b deposition. Therefore, the opsonization of SARS-CoV-2 by MBL triggers the activation of the lectin pathway and the subsequent deposition of complement factors. Moreover, they also found a relevant increase of the anaphilotoxin C5a in the sera of critically ill patients with SARS-CoV-2 infection [126,130].

Moreover, as observed for the lectin pathway, the classical pathway activation plays a pathogenic role in tissue injury. In particular, in SARS-CoV infection, the binding of autoantibodies to damaged cells mediates the complement activation, leading to further cell damage through complement-mediated cytotoxicity [131].

Studies in deficient mice for C3 have shown that the activation of C3 is essential for viral clearance and protection against infection [132]. Indeed, the absence of complement activation in these mice was associated with the development of severe bronchitis, bronchiolitis, and vasculitis, and increased inflammatory cells in the lung tissue. However, C3-deficient mice infected with SARS-CoV showed less respiratory dysfunction, and fewer lung pathology changes and inflammatory infiltrates in the lung parenchyma compared to wild-type mice [130].

The contrasting results from these studies highlight the complex functions of the complement system in viral infection. In this context, C3 inhibitors could not obtain positive effects in COVID-19 disease, since complement activation favors viral clearance [130]. Otherwise, the use of C5 inhibitors limit the C5-b9 formation and the subsequent cell damage [133,134], but also assures the C3 activation and the opsonization of SARS-CoV-2 for removal by immune cells [130].

In MERS and SARS-CoV infections, several studies have shown that local and systemic C5 activation exerts a critical role in virus-induced lung damage and mortality [130]. The role of C5 and C5a in SARS-CoV-2 infection has been described by Gao et al., who observed an improvement of clinical conditions in two patients that received multiple injections of the C5a antibody [126].

Taking into account these studies, the prevention of the complement terminal complex formation and the use of C5 inhibitors could be a promising approach to the reduction of the lung injury and systemic inflammation associated with severe forms of viral infection, such as COVID-19 disease.

## 9. Systemic Endothelial Dysfunction and Hypercoagulability in COVID-19 Disease

As has previously been described, the pathogenic mechanisms of SARS-CoV-2 infection reflect the principal hallmarks of sepsis disease [135]. Since the impairment of endothelial function plays a central role in septic shock and organ dysfunction, and has been suggested to be a predictor of mortality in sepsis [136], it is reasonable that the endothelium could be a more plausible player and target in COVID-19 disease.

The experimental studies performed by our group on sepsis-induced AKI contributed to highlight the role of the vascular compartment in systemic inflammatory diseases [16,137,138]. In the course of sepsis, we observed that endothelial cells and pericytes acquired a pro-fibrotic and dysfunctional phenotype, promoted the activation of the coagulation cascade, compromised parenchymal perfusion, and increased inflammation and fibrosis [16,137,139]. Therefore, the endothelium it is not just a passive element during sepsis, but rather actively contributes to the amplification of the inflammatory response and multi-organ damage [16].

Recent evidence supports the importance of endothelial dysfunction in COVID-19 syndrome, in addition to respiratory disease [140,141]. Varga et al. analyzed the vascular beds of several organs in COVID-19 patients, and demonstrated that the endothelial cells accumulated viral elements in their cytoplasm and promoted the recruitment of inflammatory cells, leading to endothelial dysfunction [140,141].

In another study, the histopathology analysis of kidney biopsies of deceased COVID-19 patients revealed endothelial cell swelling, and—in three of these cases—the presence of fibrin thrombus in glomerular capillary loops that was strongly associated with severe parenchymal [14,126].

As is well known, the activation of complement system is considered one of the key events in sepsis disease [142]. Its principal function for the host is to identify and neutralize the invading pathogens through the opsonization of foreign surfaces and the formation of the terminal complex C5b-9 (MAC), which leads to the lysis of the pathogen [142]. However, the activated complement exerts detrimental effects on the host endothelium through the formation of MAC on the surface membrane [142]. The dysfunctional activation of endothelial cells also occurs also during renal ischemia reperfusion injury, in which the complement activation that orchestrates the immunological and inflammatory process also controls the endothelial behavior and response [51,143,144]. Indeed, in our previous studies, we demonstrated that the complement—in particular C3a and C5a—enhanced the endothelial and pericytes to mesenchymal transition process and tubular damage, inducing vascular rarefaction and early fibrosis, with the loss of renal function [138,143,144,145,146].

The role of C5 activation products in COVID-19–associated vasculopathy is supported by several pieces of evidence that demonstrate the presence of C5b9 deposits not only in the microvasculature of lung tissue but also in many organs, including the heart and blood vessels, kidneys, gut, and brain [140].

Another important finding is that the erythrocyte aggregation in the peritubular and glomerular capillaries of the renal parenchyma in COVID-19 patients was associated with oxidative stress, inflammation and complement activation that aggravated the microvascular damage [14]. Moreover, the occlusion of the microvascular lumens by erythrocyte aggregates was responsible for several vascular lesions [14]. Even if the endothelial cells did not express ACE-2 in normal conditions, Varga et al. demonstrated that endothelitis was a common feature of organ dysfunction, and endothelial cells could be involved directly and indirectly in SARS-CoV-2 infection [88]. Interestingly, a recent study showed that SARS-CoV-2 could infect host cells through the binding of an alternative cell receptor known as CD147, which is a transmembrane glycoprotein which is highly expressed on all endothelial cells [147].

Emerging evidence has shown that SARS-CoV-2 may play an important role in inducing coagulopathy [148]. Several analyses of the hematological profile of COVID-19 patients showed high plasma levels of reactive protein C, fibrinogen, D-dimer, and ferritin, which are associated with thrombocytopenia [148]. In addition, clinical and autopsy reports from China and the U.S. have shown the presence of disseminated intravascular coagulation, with evidence of microangiopathy in several organs, including the kidneys [148]. Indeed, the activation of monocytes and macrophages, the increase of pro-inflammatory cytokines, the activation of the complement system, and the release of DAMPs can cause the activation of endothelial cells to express tissue factor (TF), and the subsequent activation of the coagulation cascade predisposing to hypercoagulability.

The hypercoagulability predisposes to the development of cortical necrosis, and therefore leads to irreversible renal damage [149].

Together, these studies point to another important rationale to find therapies that could preserve the endothelium, and inhibit viral replication, hypercoagulability, and the systemic inflammatory response.

## 10. Therapeutic Strategies in SARS-COV-2–Induced AKI

Although bacterial sepsis is not a hallmark of SARS-CoV-2 disease, the overwhelmed immune response, as previously described, underlined the similar pathological conditions of a severe CRS with multiple organ dysfunction [150]. There is no definite treatment for this complex disease, and the supportive care treatment is considered the best strategy for patients with COVID-19-induced AKI in the ICU [32].

The decrease of volume-trauma and barotrauma through the use of lung-protective ventilation prevents the occurrence of new or worsening AKI by limiting the ventilation-induced hemodynamic effects and the cytokine cargo on the kidney [151]. Another strategy is to regulate the fluid balance according to volume responsiveness and tolerance assessment. In this way, normal volume status could be restored in order to avoid volume excess and reduce the risk of pulmonary edema, right ventricular overload, congestion, and subsequent AKI [87].

If the standard care treatment fails, renal replacement therapy should be considered an adequate treatment in order to assure organ support and prevent AKI. In particular, blood purification could be considered the best option to restore ‘immune homeostasis’ in this complex disease, which is characterized by a dysregulated host response to infection and consequent multiple organ dysfunction [87]. Extracorporeal blood purification is proposed as an adjuvant therapy for COVID-19 disease, aiming at controlling the associated dysregulation of the immune response through the removal of increased cytokines and chemical mediators [87]. This approach comprises a group of techniques including plasma exchange, adsorption, perfusion, and continuous renal replacement therapy (CRRT) [138,152].

Among them, CRRT could be considered the best strategy for severe forms of COVID-19 characterized by AKI, systemic inflammatory response syndrome, multiple organ dysfunction syndrome, and CRS. Indeed, the emerging literature highlights that the number of patients with AKI requiring CRRT is unprecedented in modern history [53]. These therapeutic interventions have proved fundamental as lifesaving measures in patients with severe forms of COVID-19 and impaired renal function [153,154,155].

Park et al. [156] showed that CRRT reduced the serum levels of IL6 and IL8 in septic disease. Moreover, a randomized clinical trial underlined that the early use of CRRT could significantly reduce the index of mortality in critically ill patients with renal damage [157]. All of these studies demonstrate that the timely use CRRT may be effective in reducing the highest peak of the cytokine storm in severe forms of COVID-19.

In April 2020, the Food and Drug Administration (FDA) temporarily approved the use of adsorption/CytoSorb therapy in the management of CRS in COVID-19 patients [158,159]. Considering several clinical studies on Cytosorb performance, the FDA permitted the use of this adsorbent membrane in COVID-19 disease in order to reestablish the equilibrium between pro- and anti-inflammatory mediators, and it is considered to be a ‘salvage,’ or ‘compassionate use’ intervention when other pharmacological treatments are ineffective [87].

A controlled trial has been conducted on critically ill COVID-19 patients, showing the efficacy of cartridges in adsorbing inflammatory cytokines such as IL-6, and in improving the general conditions in most of recruited patients [160]. In a case report, extracorporeal blood purification has been proven to effectively remove the released inflammatory cytokines and to improve renal function in terms of serum creatinine and urine output [161].

Certainly, the nonspecific or specific removal of some DAMPS or pathogen-associated molecular pattern molecules (PAMPs) plays a key role to counter the systemic peaks of cytokine concentrations, which are disproportionate and counterproductive in severe inflammatory diseases, such as COVID-19. However, patients differ from each other based on their inflammatory phenotype, and they develop different levels of cytokines in their blood [162].

To our knowledge, there is a paucity of data concerning the effects on mortality and favorable outcomes after AKI of CRRT versus other therapeutic strategies. Given the rapid continuous expansion of COVID-19 literature, many cohort studies regarding the management of AKI in the setting of COVID-19 had limitations in their description of the details of CRRT and other treatments.

Recently, Wilbers et al. investigated the mortality rate and renal recovery in patients with AKI and RRT due to COVID-19. They showed a comparable mortality index in the RRT versus non-RRT group in a retrospective cohort study. In addition, neither group required RRT after ICU discharge [163]. In another retrospective cohort study, Yang et al. analyzed COVID-19 patients undergoing invasive mechanical ventilation, comparing patients who received CRRT treatment and those treated with conventional standard therapies (without CRRT) [164]. Interestingly, CRRT was associated with prolonged survival in COVID-19 patients [164].

In an in-depth review, Ronco et al. indicated practical recommendations for CRRT in COVID-19 patients, highlighting several criteria including clinical conditions, laboratory parameters, and AKI biomarkers, which are useful when defining the effective sequential extracorporeal therapies in clinical practice to avoid renal damage. Further research is needed in order to establish the importance of the early initiation of RRT and sequential extracorporeal therapies in the management of COVID-19 patients.

## 11. Conclusions

In brief, there is a high incidence of AKI in critical ill patients with COVID-19. The exact mechanism of this renal impairment has not yet been clarified. In the absence of specific treatments, supportive care and blood purification techniques for critically ill patients with renal injuries are considered to be the best approaches both for clinical management and to increase the probability of a favorable outcome.

Because the mortality rate of critically ill SARS-CoV-2 patients with AKI is three times higher than for those without AKI, it is important to diagnose AKI in a timely fashion. Increasing the knowledge of the pathogenesis of COVID-19 associated AKI could suggest targeted interventions that should modulate the severity of kidney failure and significantly improve the clinical outcomes of COVID-19 patients.
